# The Role of NF-κB and Bax/Bcl-2/Caspase-3 Signaling Pathways in the Protective Effects of Sacubitril/Valsartan (Entresto) against HFD/STZ-Induced Diabetic Kidney Disease

**DOI:** 10.3390/biomedicines10112863

**Published:** 2022-11-09

**Authors:** Mohamed Mohany, Mohammed M. Ahmed, Salim S. Al-Rejaie

**Affiliations:** Department of Pharmacology and Toxicology, College of Pharmacy, King Saud University, P.O. Box 55760, Riyadh 1145, Saudi Arabia; mmohany@ksu.edu.sa (M.M.); mmahmed114@yahoo.com (M.M.A.)

**Keywords:** type 2 diabetes, diabetic kidney disease, inflammation, apoptotic markers, LCZ696

## Abstract

LCZ696 (valsartan/sacubitril) has the potential to slow the progression of diabetic kidney disease (DKD) according to previous reports. However, the renoprotective mechanism underlying LCZ696 remains unknown. This study aimed to investigate the therapeutic potential and underlying mechanism of LCZ696 in DKD in a type 2 diabetic (T2D) rat model. This model was established in this experiment by feeding a high-fat diet (HFD) for six weeks with a single dose of streptozotocin (STZ, 30 mg/kg body weight). Valsartan or LCZ696 was orally administered to T2D animals for eight weeks. HFD/STZ rats showed hyperglycemia, impaired insulin secretion, significant increases in urea, creatinine, cytokines, nuclear factor kappa B (NF-κB), oxidative stress, caspase-3 activity, glomerular and tubular damage, glomerulsclerosis, Bax and caspese-3 expressions along with a significant decline in IL-10, antioxidant markers, and Bcl-2 expression. The administration of LCZ696 to diabetic rats reduced the serum concentrations of glucose, urea, and creatinine. In addition, ELISA results demonstrated that diabetic rats treated with LCZ696 exhibited a reduction in inflammatory (IL-1β, TNF-α, IL-6) and an increase in anti-inflammatory (IL-10) cytokine levels. In addition, a notable decrease in NF-κB and caspase-3 activity was observed. At the level of renal tissue homogenate, diabetic animals treated with LCZ696 demonstrated clear restorations in GSH content and other antioxidant enzyme levels, in addition to a significant decrease in TBARS levels. In addition, LCZ696 inhibited the expression of the Bax and cleaved caspase-3 proteins and enhanced the expression of the Bcl-2 protein. Improvements in histopathological changes in kidney tissues confirmed and significantly supported these biochemical findings. In summary, LCZ696 alleviated DKD with possible mechanisms including inhibition of inflammation and apoptosis.

## 1. Introduction

Globally, more than 462 million individuals worldwide suffer from type 2 diabetes mellitus (T2DM), which is the most common kind of chronic disease [1]. Insulin resistance and a gradual loss of β-cell function that results in hyperglycemia are the major features of T2DM [2]. Without strict management, hyperglycemia can damage organs and result in a range of significant side effects, such as retinopathy, neuropathy, and nephropathy [3]. One of the most common complications of diabetes is diabetic kidney disease (DKD), which is the primary cause of end-stage renal disease (ESRD) [4]. Type 2 diabetes mortality is predominantly caused by patients with kidney disease according to previous reports [5]. Albuminuria and glomerular hyperfiltration are symptoms of the early stages of DKD, whereas a progressive deterioration in renal function as demonstrated by altered serum creatinine is a symptom of the late stages [6]. Some DKD patients also show substantial interstitial and vascular fibrosis on the renal histology in addition to these symptoms [7]. The development and progression of DKD are largely dependent on persistent renin–angiotensin–aldosterone system (RAAS) activation caused by chronic hyperglycemia. Currently, blood pressure control, RAAS suppression, and hyperglycemia control are the cornerstones of DKD care [8].

It is well established that inflammation and oxidative stress play an axial role in DKD initiation and progression [9,10]. Therefore, by controlling these two pathways, DKD development could be managed or stopped. The imbalance between the generation of reactive oxygen species (ROS) during hyperglycemia and antioxidant defense in cells can result in tissue injury through necrosis and ultimately apoptosis is what causes an oxidative stress state [11]. The most severe damage to any cellular components is brought on by oxidative stress, which also damages DNA, cellular proteins, and lipids [12]. Numerous investigations on the inflammatory response in DKD have shown that proinflammatory cytokines such as tumor necrosis factor-α (TNF-α), interleukin-1β (IL-1β), and interleukin-6 (IL-6) are present at higher levels in both DKD patients [13] and experimental animal models [14]. The activation of crucial transcription factors like the nuclear factor kappa B (NF-κB), which promotes apoptotic, fibrotic, and inflammatory processes that play a significant role in cell injury and associated issues, is one of the most crucial effects of hyperglycemia-induced oxidative stress [15]. Consequently, inhibiting NF-κB signaling pathway can help with the control of vascular complications of diabetes [16]. Apoptosis has been identified as a significant contributor to renal fibrosis and is one of the distinctive morphologic alterations seen in DKD [17]. As a result, inhibiting apoptosis is a useful tactic for reducing the effects of DKD.

The combination of valsartan and sacubitril, known as LCZ696, an angiotensin receptor–neprilysin inhibitor, lowers morbidity and mortality in patients with heart failure [18]. LCZ696 functions by minimizing the activity of the RAAS and enhancing the cardiovascular defense offered by the natriuretic peptide system [19]. We recently discovered that LCZ696 therapy can slow the evolution of diabetic nephropathy by preventing oxidative stress, inflammation, and glomerulosclerosis in hyperglycemic rats [20]. Clinical trials and other experimental research showed that LCZ696 had therapeutic benefits for diabetes patients [21,22,23], prevented proteinuria in diabetic rats [24], and prevented DKD in *db/db* mice [25]. However, there is a dearth of information on its advantages and how it affects type 2 diabetes-induced DKD. Therefore, the goal of the current experiment is to investigate the potential protective benefits and mechanisms of LCZ696 against DKD in a well-established rat model of experimental type 2 diabetes using a high-fat diet and streptozotocin (HFD/STZ).

## 2. Materials and Methods

### 2.1. Chemicals and Drugs

STZ was purchased from Sigma-Aldrich, Inc., (St. Louis, MO, USA), valsartan, Tabuvan^®^ 80 mg from (Tabuk pharmaceutical Manufacturing Co., Tabuk, Saudi Arabia), and LCZ696 (sacubitril/valsartan, Entresto™ 200 mg from Novartis Pharma AG, Basel, Switzerland). All other chemicals and reagents were of the highest analytical grade commercially available.

### 2.2. Animal Care and Ethical Approval

Adult male Wistar rats weighing between 100 and 120 g were collected from the Experimental Animal Care Center at the Pharmacy College of King Saud University, where they were cared for and monitored in a particular pathogen-free environment. All experimental procedures, including euthanasia, were carried out following ethical standards established by the Experimental Animal Care Centre at King Saud University (KSU), Riyadh, Kingdom of Saudi Arabia (ethics reference no: SE-19-118) and the National Institute of Health Guide for the Care and Use of Laboratory Animals, Institute for Laboratory Animal Research (NIH publications no. 80–23; 1996). Before research, all animals were given 7 days to acclimate in polycarbonate cages in a well-ventilated environment. Standard laboratory settings (temperature of 23–24 °C, relative humidity of 60–70%, and a 12 h light/dark cycle) were used to sustain the animals.

### 2.3. Induction of Type 2 Diabetes in Experimental Animals

T2D was established in rats as previously described [26]. HFD feeding for 6 weeks (D12451, 45 kcal% fat manufactured by Research Diets Inc., New Brunswick, NJ, USA) and a single low dosage of STZ (30 mg/kg body weight) dissolved in freshly prepared citrate buffer (0.1 M, pH 4.5) administered intraperitoneally (i.p.) resulted in the induction of type 2 diabetes in rats. Then, estimating fasting blood glucose levels from the tail vein of diabetic animals was done two days after STZ injection using a glucometer (Accu-chek compact-plus glucose meter system) (Roche Diagnostics, Meylan, France). Animals with glucose levels exceeding 250 mg/dL were considered diabetic and used in the experiment. Throughout the experiment, the control group was fed a D12450H control diet (10 kcal% fat, manufactured by Research Diets Inc., NJ, USA) and citrate buffer (0.1 M, pH 4.5) was injected i.p. as a vehicle.

### 2.4. Experimental Procedures

**Figure 1:**
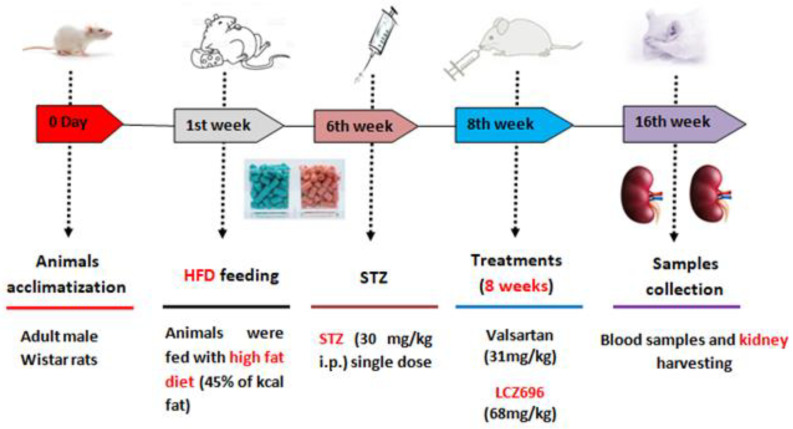
Diagram illustrating the experimental design.

The study plan for the current experiment is presented in Figure 1. After inducing diabetes, rats were randomly assigned to one of four groups (*n* = 8): (1) normal rats treated with vehicle (control), (2) HFD/STZ rats treated with vehicle (HFD/STZ), (3) HFD/STZ rats treated with valsartan (valsartan), and (4) HFD/STZ rats treated with LCZ696 (LCZ696). Valsartan, Tabuvan^®^ 80 mg (Tabuk pharmaceutical Manufacturing Co, Tabuk, KSA), was suspended in 0.5% carboxymethylcellulose (CMC) and supplied at a dose of 31 mg/kg/day through gastric gavage to normal and diabetic rats at approximately the same time each day. LCZ696 (sacubitril/valsartan, EntrestoTM 200 mg, NOVARTIS, Switzerland) was suspended in 0.5% carboxymethylcellulose (CMC) and delivered through gastric gavage to normal and diabetic rats at a dose of 68 mg/kg/day roughly every day at the same time. The treatment began two weeks after the onset of diabetes and continued for eight weeks. On the basis of our earlier research and other previous reports [26,27], we determined the course of treatment and the valsartan and LCZ696 dosages. The body weights of animals were recorded weekly on the same day and around the same time. The animals were fasted overnight and anesthetized with a mixture of ketamine (Hikma Pharmaceuticals, Jordan, 100 mg/kg) and xylazine (Laboratories Calier, Spain, 10 mg/kg) following the experimental period. Blood samples were extracted from the heart, placed in clean tubes, separated by centrifugation at 3000 rpm (800 g) for 10 min, and then frozen at −80 °C until analysis. Both kidneys were dissected, weighed, and the final weight was determined in grams per 100 g of body mass. A small portion of each animal’s kidney was immersed and fixed in 10% neutral buffer formalin (pH 7.4) for subsequent histopathological and immunohistochemical examinations. The remaining portion of the kidney samples was immersed in liquid nitrogen for one minute and then frozen at −80 °C until analysis.

### 2.5. Biochemical Tests of Serum

Glucose levels in serum were measured using a commercially available kit (Randox Laboratories Ltd., Crumlin, UK and SPI bio, Montigny le Bretonneux, France). ELISA was utilized to measure insulin levels (Merck Millipore, Burlington, MA, USA). The levels of creatinine and urea were measured using colorimetric techniques (Linear Chemicals, Barcelona, Spain). The serum concentrations of NF-κB, IL-1β, TNF-α, IL-6, and IL-10 were measured using ELISA techniques (Thermo Scientific, Rockford, IL, USA).

### 2.6. Tissue Analysis

Small portions of the kidneys were homogenized in a physiological buffer (1:10, *w*/*v*) and total protein concentrations were measured according to Lowry assay (1951) [28] using bovine serum albumin as a standard. TBARS and GSH levels were measured by using ELISA kits (Cayman Chemical Co., Ann Arbor, MI, USA). In Post-mitochondria supernatants of kidney samples, enzymatic activities of SOD, CAT, GPx, and GST were measured by using ELISA kits (R&D systems Inc., Minneapolis, MN, USA).

### 2.7. Caspase-3 Assay

According to the manufacturer’s instructions, the caspase-3 colorimetric assay kit (R&D Systems, Minneapolis, MN, USA) measured the increased enzymatic activity of the caspase-3 class of proteases in renal tissue. In a 96-well microplate, the enzymatic reaction for caspase activity was performed by adding 250 μg protein/50 μL of rat kidney homogenate. The cleavage of caspase-3 colorimetric substrate (DEVD-pNA) by caspase releases the chromophore pNA, which was measured spectrophotometrically at 405 nm using a microplate reader (ELx800 Absorbance Reader, BioTek Instruments Co., Winooski, VT, USA) The results are expressed as a fold increase in caspase activity, as indicated by an increase in optical density.

### 2.8. Renal Histological Evaluation

The kidneys that were harvested were fixed in 10% formalin, dehydrated, and then embedded in paraffin wax. The sections (5–7 m) were cut with an automated microtome (Leica RM 2125 RM, Leica Microsystems, Nussloch, Germany) and mounted on glass slides. All slides were stained with hematoxylin and eosin (H&E) and periodic acid–Schiff (PAS) and examined by an experienced pathologist using a light microscope, the Nikon Eclipse E600, and a digital high-resolution camera. Briefly, 20 glomeruli per section were scored to determine the average glomerular injury index per rat, with a score of 0 for intact glomeruli, 1 for less than 25%, 2 for 25–50%, 3 for 50–75%, and 4 for >75% damage. The score for tubular damage was as follows: 0 = normal histology; 1 = tubular cell enlargement, brush border loss, nuclear condensation, and up to one-third nuclear loss; 2 = as in 1, but greater than one-third and less than a two-thirds nuclear loss in tubules; and 3 = greater than two-thirds nuclear loss. Each group had three randomly selected visual fields evaluated blindly by two researchers. Kidney sections stained with PAS were utilized in a glomerulosclerosis demonstration in which 20 glomeruli were randomly selected from each section and positive signals within the selected glomerulus were highlighted, measured, and represented as a percentage of the entire glomerulus’ positive area as previously described [29].

### 2.9. Immunohistochemistry

Using the avidin–biotin–peroxidase complex (ABC) technique, immunohistochemistry was performed on paraffin sections mounted on positively charged slides. This IHC examination utilized the following antibodies: Rabbit Anti-caspase-3 antibody active (cleaved) form, monoclonal, Sigma Aldrich Cat# AB3623; Rabbit Anti Bcl-2 antibody, polyclonal, Abcam Cat# ab196495; and Mouse Anti Bax antibody, monoclonal, Sigma Aldrich Cat# B84293. After incubating each group’s sections with the previously mentioned antibodies, the ABC method reagents (Vectastain ABC-HRP kit, Vector laboratories) were added. To detect the antigen–antibody complex, marker expression was labeled with peroxidase and colored with diaminobenzidine (DAB, manufactured by Sigma). Negative controls were implemented by substituting nonimmune serum for the primary or secondary antibodies. Sections stained with IHC were examined using a Leica microscope (CH9435 Hee56rbrugg) (Leica Microsystems, Switzerland). Using the Leica Qwin 500 image analyzer computer system (Cambridge, UK), the area percentage of immunohistochemically positive structures was quantified.

### 2.10. Data Analysis

Data are presented as mean and standard error (mean ± SEM). One-way ANOVA was performed to test the significant differences between groups using GraphPad Prism version 8 (GraphPad Software, Inc., La Jolla, CA, USA). Tukey, a multiple comparison test, was utilized as a post hoc test. Statistical significance was set at *p* < 0.05.

## 3. Results

### 3.1. Effects of LCZ696 and Valsartan on Serum Glucose, Insulin, and Renal Functions in HFD/STZ-Induced Rats

Serum biochemical analysis showed a significant increase in blood glucose levels in HFD/STZ-induced rats (*p* < 0.001) in comparison with the control group, and these levels were significantly reduced in diabetic animals treated with LCZ696 (*p* < 0.01) and valsartan (*p* < 0.05). Although HFD/STZ-induced rats showed lower blood insulin levels than control rats, there were no significant differences in insulin levels between the different groups. Urea and creatinine were significantly higher in the serum of HFD/STZ-induced rats compared with the control group. When compared with the HFD/STZ-induced rats, the LCZ696 treatment significantly decreased serum urea (*p* < 0.01) and creatinine levels (*p* < 0.001). Additionally, compared with the valsartan group, LCZ696 treatment of diabetic rats had a greater reduction in serum urea and creatinine (Table 1).

### 3.2. Lcz696 Reduced Inflammatory State in Hfd/Stz-Induced Rats

LCZ696 was tested for its anti-inflammatory effects on diabetic rats by measuring serum cytokine levels (IL-6, IL-1β, TNF-α, and IL-10) (Table 1). The circulating levels of proinflammatory cytokines (TNF-α, IL-1β, and IL-6) cytokines were significantly (*p* < 0.001) elevated in HFD/STZ-induced rats, and treatment with LCZ696 or valsartan reversed these cytokines with LCZ696 having a stronger effect on IL-1β compared with the valsartan group. In contrast, HFD/STZ-induced rats had significantly lower serum levels of IL-10 (*p* < 0.01) than the control group. Intriguingly, animals treated with LCZ696 or valsartan showed a significant rise in the serum level of IL-10 when compared with diabetic animals.

### 3.3. LCZ696 Attenuated HFD/STZ-Induced Activation of NF-Kb (P65) and Caspase 3 Via Inhibiting NF-Kb Signaling and Caspase 3 Signaling

**Figure 2:**
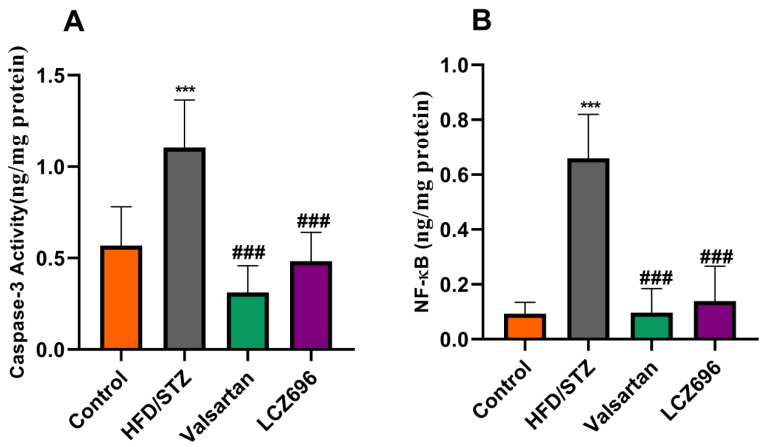
The effects of LCZ696 and valsartan treatment on caspase-3 activity (**A**) and NF-κB (**B**) in HFD/STZ-induced diabetic rats. The data are expressed as the mean ± SEM, *n* = 6, *** *p* < 0.001 versus control; ^###^ *p* < 0.01 versus HFD/STZ. Abbreviations: NF-κB, nuclear factor kappa-B.

NF-k (p65) and caspase-3 activity were assessed by spectrophotometry to support the hypothesis that HFD/STZ caused inflammation, cellular damage, and death. According to the current investigation, NF-k (p65) and caspase-3 activities in the kidney tissue homogenate of HFD/STZ-induced rats were significantly increased (*p* < 0.001) when compared with the control group. After LCZ696 and valsartan administration to diabetic animals, these high levels of NF-k (p65) and caspase-3 activity in HFD/STZ-induced rats dramatically (*p* < 0.001) decreased (Figure 2).

### 3.4. LCZ696 Alleviated Oxidative Stress and Enhanced Antioxidants in HFD/STZ-Induced Rats

**Figure 3:**
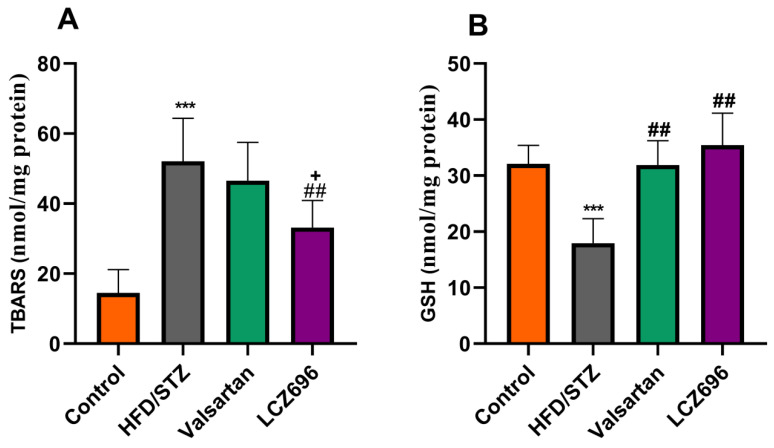
The effects of LCZ696 and valsartan administration on renal oxidative stress markers in HFD/STZ-induced diabetic rats, including thiobarbituric acid reaction substances (TBARs) (**A**) and glutathione (GSH) (**B**). The data are expressed as the mean ± SEM, *n* = 6, *** *p* < 0.001 versus control; ^##^ *p* < 0.01 versus HFD/STZ; ^+^ *p* < 0.05 versus valsartan.

**Figure 4:**
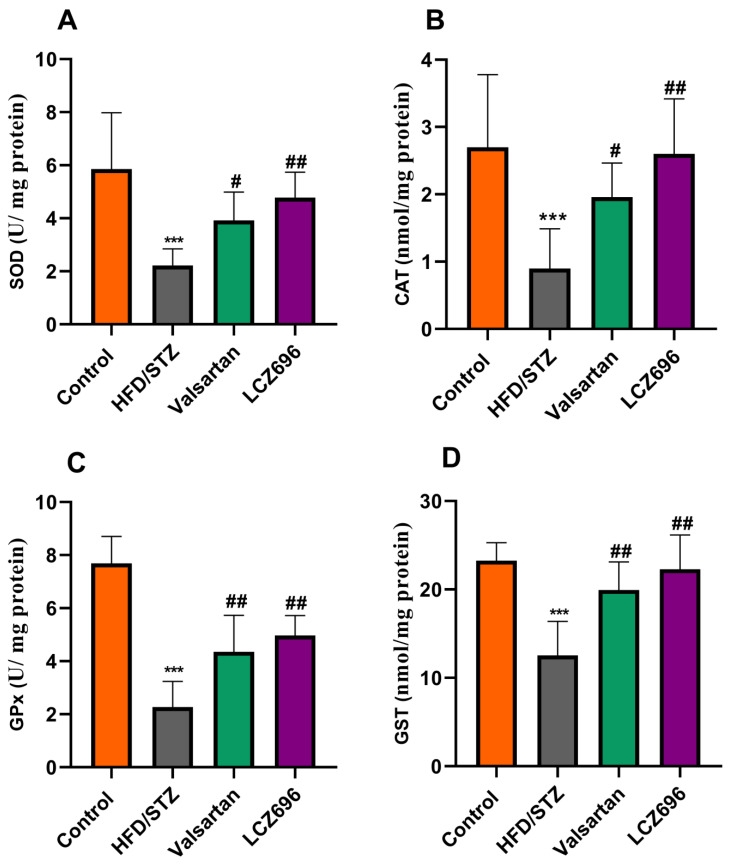
The impact of LCZ696 and valsartan treatment on the renal antioxidant status of HFD/STZ-induced diabetic rats, including superoxide dismutase (SOD) levels (**A**), catalase (CAT) activity (**B**), glutathione peroxidase (GPx) (**C**), and glutathione-S-transferase (GST) (**D**). The data are expressed as the mean ± SEM, *n* = 6, *** *p* < 0.001 versus control; ^#^ *p* < 0.05, ^##^ *p* < 0.01 versus HFD/STZ.

Analyses of renal TBARs and antioxidant enzymes (Figure 3 and Figure 4) were conducted on diabetic animals to assess LCZ696’s protective effects on oxidative stress. HFD and STZ-treated rats exhibited renal oxidative stress as indicated by significantly higher (*p* < 0.001) TBARs than control rats. HFD/STZ-induced rats also showed significant reductions (*p* < 0.001) in renal GSH, SOD, CAT, GPx, and GST activities. Treatment with LCZ696 significantly reduced (*p* < 0.01) the high levels of renal TBARs by 36.42% compared with the HFD/STZ group, and by 28.82% when compared with the valsartan group. Additionally, LCZ696 or valsartan administration improved renal GSH, SOD, CAT, GPx, and GST activities compared with the HFD/STZ group, and there was a trend toward higher antioxidant enzymes compared with valsartan administration.

### 3.5. LCZ696 Prevents Kidney Damage in HFD/STZ-Induced Rats

**Figure 5:**
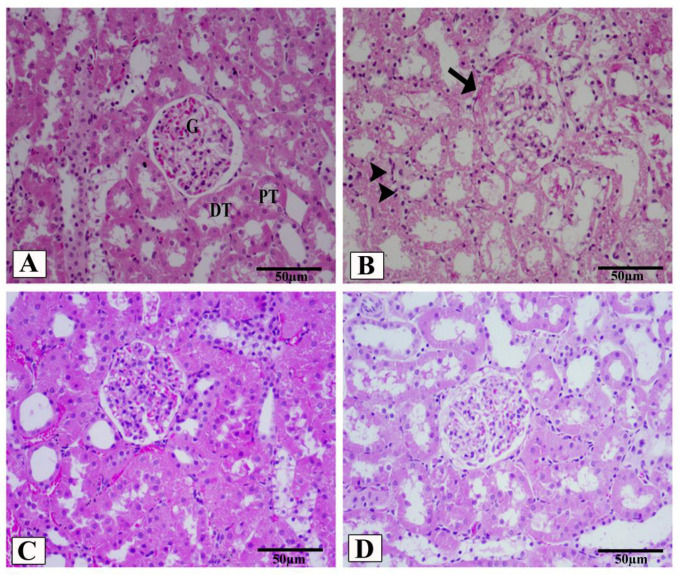

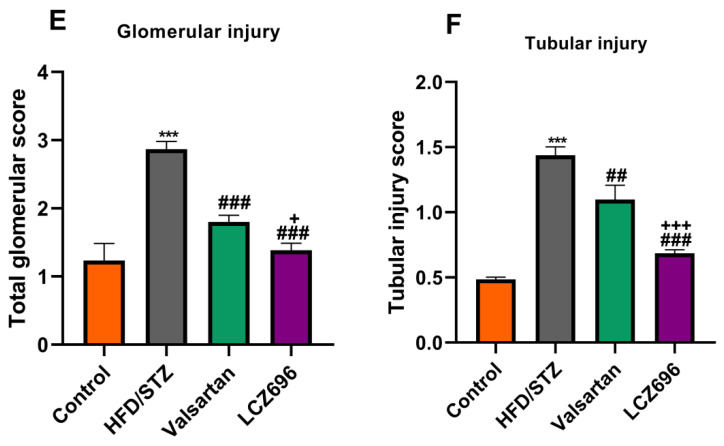
Renal cortex photomicrographs of H&E-stained kidney sections from control (**A**), HFD/STZ-induced rats (**B**), valsartan-treated group (**C**), and LCZ696-treated animals (**D**). The renal cortex of the control group displays normal proximal convoluted tubules (PT), distal convoluted tubules (DT), Bowman’s capsule, and glomerulus structures (G). The renal cortex of animals given HFD/STZ exhibited enlargement of the urinary space, thickening of the basal membrane of the glomerulus (arrow), and infiltration of inflammatory mononuclear cells (head arrow). Glomeruli and renal tubules were significantly improved in the renal cortex of HFD/STZ rats treated with valsartan and LCZ696. Damages to the glomeruli (**E**) and tubules (**F**) in the kidneys of rats from different groups were assessed semi-quantitatively, data are presented as mean ± SEM, *n* = 6, *** *p* < 0.001 versus control; ^###^ *p* < 0.001 and ^##^ *p* < 0. 01 versus HFD/STZ; ^+^ *p* < 0.05 and ^+++^ *p* < 0.001 versus valsartan. H&E, scale bar = 50 µm.

**Figure 6:**
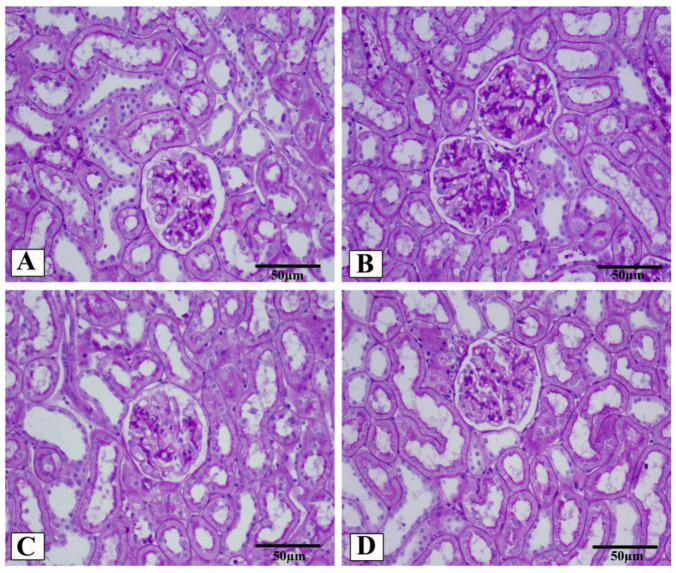

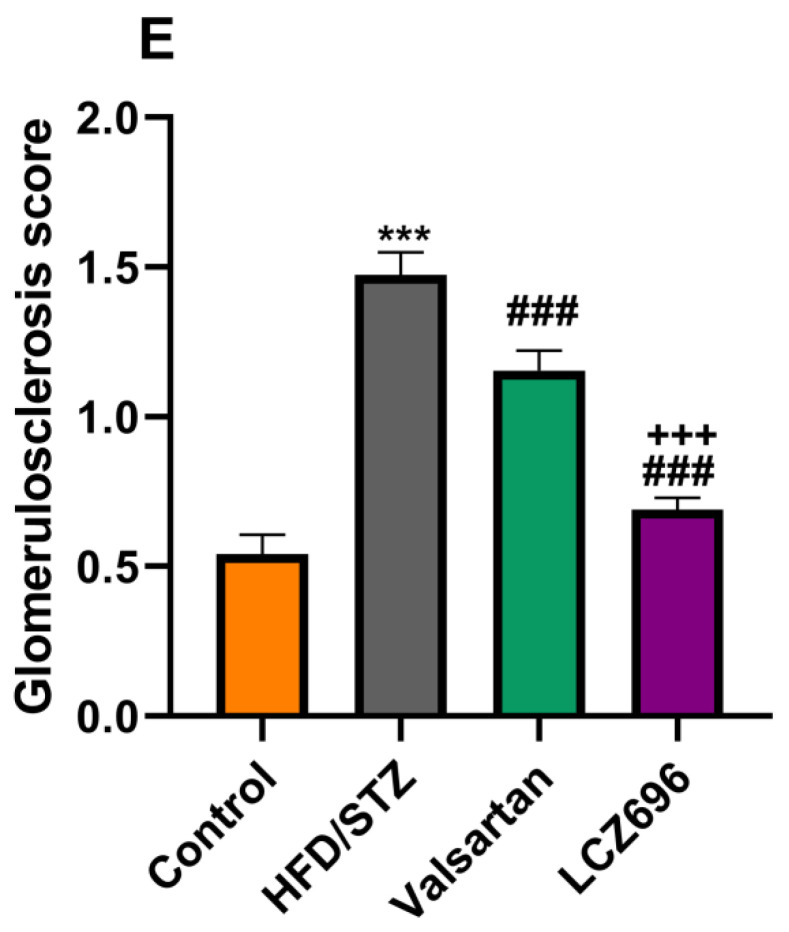
Representative photomicrographs of glomeruli stained with periodic acid–Schiff (PAS, scale 50 µm) from control (**A**), HFD/STZ (**B**), valsartan-treated group (**C**), and LCZ696-treated animals (**D**). The dark purple color in the glomerulus is the extracellular matrix. In different groups, the area of the glomerular matrix was measured (**E**). Data are presented as mean ± SEM, *n* = 6, *** *p* < 0.001 versus control; ^###^ *p* < 0.001 versus HFD/STZ; ^+++^ *p* < 0.001 versus valsartan.

**Figure 7:**
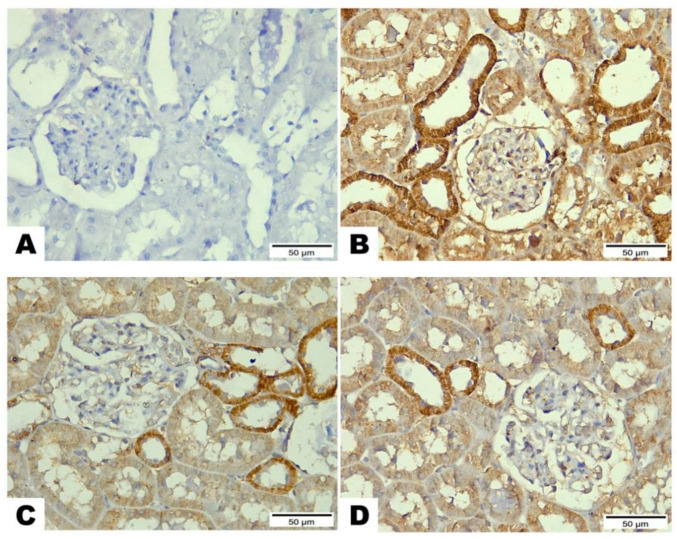

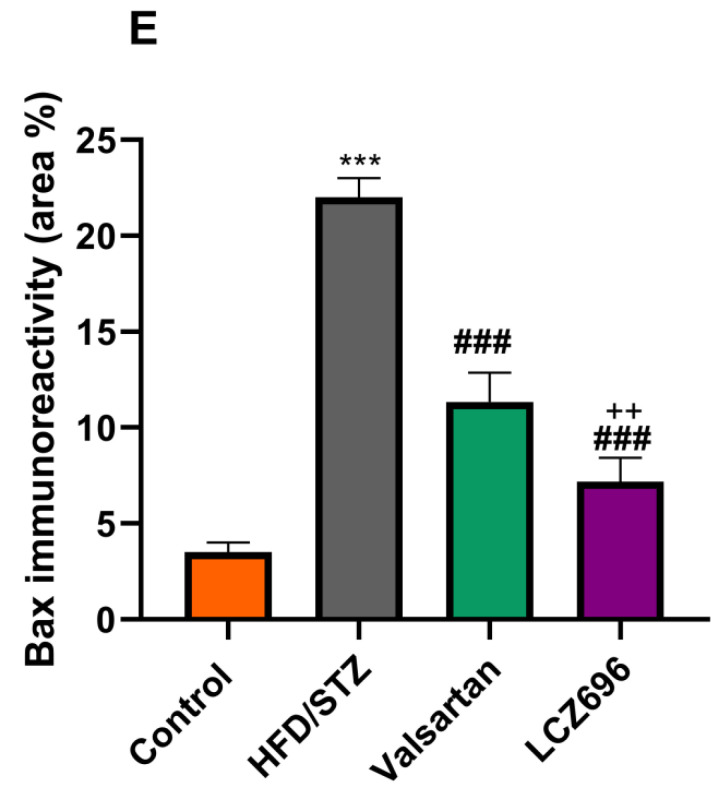
The effects of LCZ696 or valsartan on renal apoptosis marks in HFD/STZ-induced diabetic rats. A representative photomicrograph of renal cortex showing Bax immunohistochemistry protein distribution in different study groups. (**A**) control, (**B**) HFD/STZ, (**C**) valsartan-treated group, and (**D**) LCZ696- treated group. Area percent of immunoreactivity of Bax were quantified (**E**), (*n* = 3); data are presented as mean ± SEM, *** *p* < 0.001 versus control; ^###^ *p* < 0.001 versus HFD/STZ; ^++^ *p* < 0.01 versus valsartan (scale bar 50 µm).

In the present study, kidney histological morphology changes were evaluated using H&E and periodic acid–Schiff (PAS) staining (Figure 5 and Figure 6). H&E staining revealed that HFD/STZ-induced diabetic rats exhibited severe glomerular and tubular injuries, as evidenced by an enlarged urinary space, thickening of the glomerular capillary basement membrane, infiltration of inflammatory cells, tubular dilatation, and disordered, as well as an enlarged, mesangial area. PAS staining revealed increased glomerulus, thickened glomerular basement membrane, and glycogen accumulation in HFD/STZ-induced rats. Interestingly, after eight weeks of treatment with valsartan and LCZ696, all renal histological changes induced by HFD/STZ were significantly attenuated in the treated groups, as evidenced by decreased glomerular and tubular damage (*p* < 0.01 or *p* < 0.001), respectively. Compared with valsartan, LCZ696 treatment improved glomeruli and renal tubules significantly (*p* < 0.05 or *p* < 0.001). Using PAS-stained sections, the percentage of glomerulosclerosis (Figure 7) in HFD/STZ-induced rats was determined. In the HFD/STZ group, glomerulosclerosis was significantly (*p* < 0.001) more prevalent than in the control group. Intriguingly, treatment with valsartan and LCZ696 effectively prevented the progression of glomerulosclerosis in HFD/STZ-induced animals, with LCZ696 being more effective than valsartan in reducing glomerular matrix area.

### 3.6. Immunohistochemical Analysis of Bcl-2, Bx, and Caspase-3 after LCZ696 or Valsartan Treatment of HFD/STZ-Induced Rats

**Figure 8:**
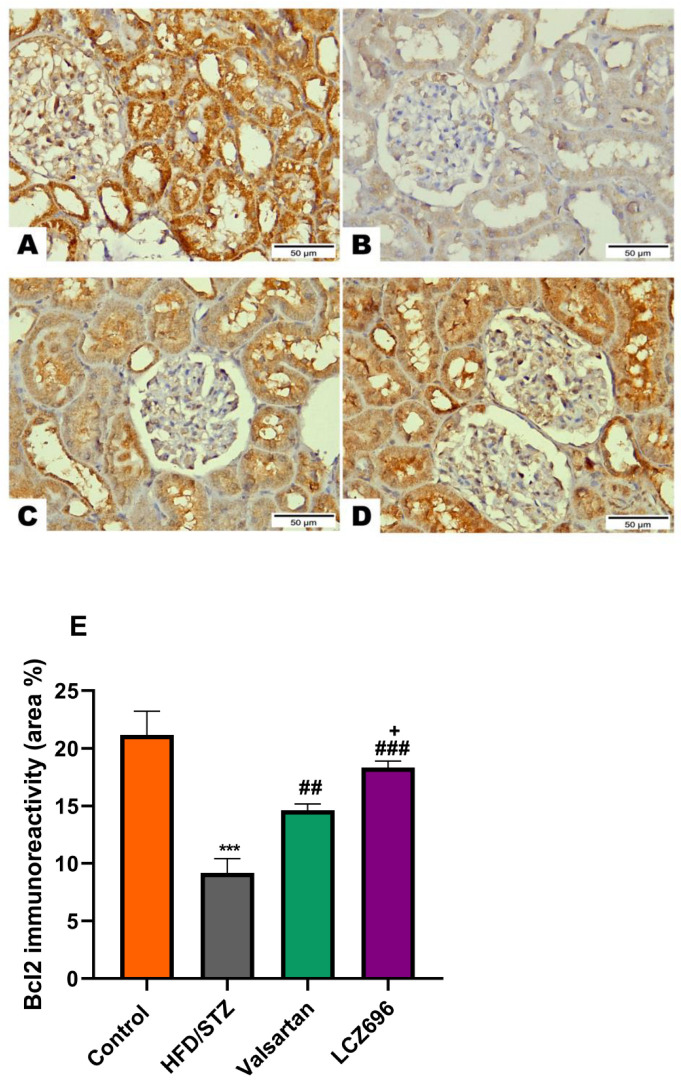
The effects of LCZ696 or valsartan on renal apoptosis marks in HFD/STZ-induced diabetic rats. A representative photomicrograph of renal cortex showing Bcl-2 immunohistochemistry expression in different study groups. (**A**) control, (**B**) HFD/STZ, (**C**) valsartan-treated group, and (**D**) LCZ696-treated group. Area percent of immunoreactivity of Bcl-2 were quantified (**E**), (*n* = 3); data are presented as mean ± SEM, *** *p* < 0.001 versus control; ^##^ *p* < 0.01 and ^###^ *p* < 0.001 versus HFD/STZ; ^+^ *p* < 0.01 versus valsartan (scale bar 50 µm).

**Figure 9:**
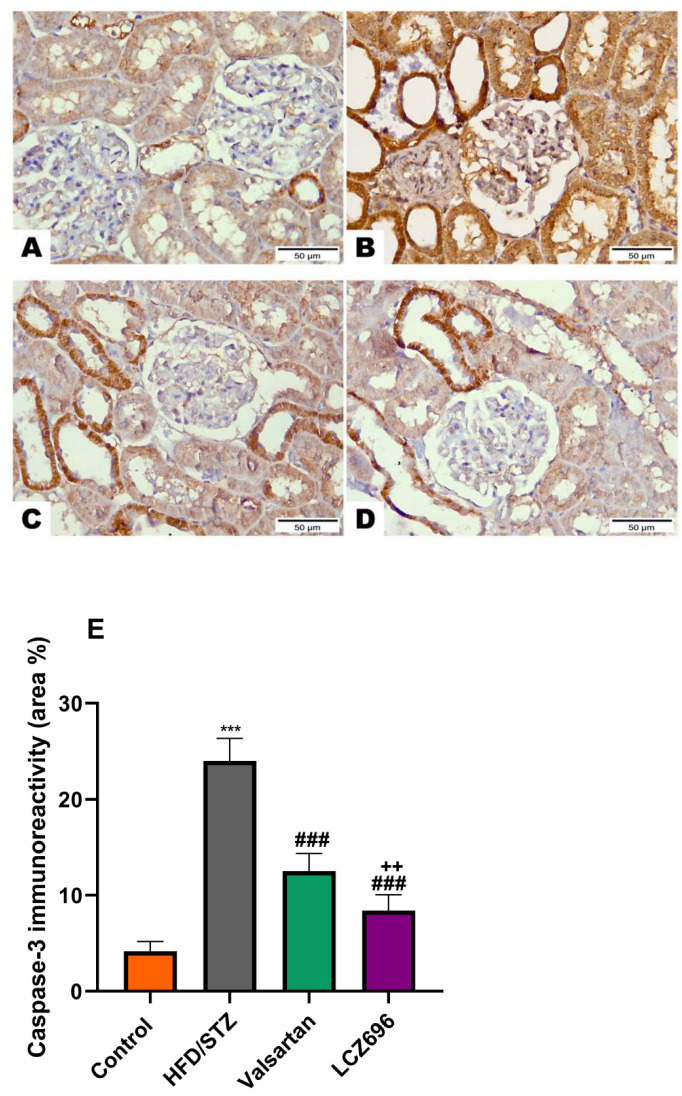
The effects of LCZ696 or valsartan on renal apoptosis marks in HFD/STZ-induced diabetic rats. A representative photomicrograph of renal cortex showing caspase-3 immunohistochemistry expression in different study groups. (**A**) control, (**B**) HFD/STZ, (**C**) valsartan-treated group, and (**D**) LCZ696- treated group. Area percent of immunoreactivity of caspase-3 were quantified (**E**), (*n* = 3); data are presented as mean ± SEM, *** *p* < 0.001 versus control; ^###^ *p* < 0.001 versus HFD/STZ; ^++^ *p* < 0.01 versus valsartan (scale bar 50 µm).

Based on the immunohistochemical analysis in the current study, the HFD/STZ group’s Bax expression in the proximal and distal renal tubule cells dramatically increased (*p* < 0.001) while being treated with valsartan or LCZ696 significantly decreased (*p* < 0.001) showing weak expression. Additionally, in HFD/STZ-induced rats, LCZ696 therapy also dramatically reduced (*p* < 0.01) the Bax protein distribution compared with the valsartan-treated group (Figure 7). Contrarily, renal tubule sections from the HFD/STZ group revealed a significantly lower level of Bcl-2 expression (*p* < 0.001) than those from control rats, while LCZ696 or valsartan treatment markedly increased this expression and LCZ696 effect was superior to that of valsartan treatment (*p* < 0.05) (Figure 8). According to the current immunohistochemistry data, LCZ696 can prevent the apoptosis that is brought on by HFD/STZ in renal tissues. Similarly to Bax expression, quantitative analysis by densitometry revealed significant increases (*p* < 0.001) in the expression of caspase-3 in the renal tissue for rats treated with HFD/STZ in comparison with sections of control rats. When valsartan or LCZ696 were administered, these alterations were significantly alleviated (*p* < 0.001) by producing weak caspase-3 reactions. HFD/STZ-induced diabetic rats treated with LCZ696 showed a greater reduction (*p* < 0.01) in caspase-3 than those treated with valsartan (Figure 9).

## 4. Discussion

DKD, which is regarded as the primary cause of ESRD, is one of the most frequent complications of type 2 diabetes [30]. Recently, there has been a growing interest and a substantial number of reports showing that Angiotensin receptor–neprilysin inhibitors (ARNIs) are beneficial for diabetic complications [31,32]; however, the precise mechanism is still not fully known. Herein, the current study is a follow-up to a previous one in which we demonstrated that therapy with LCZ696 reduced oxidative stress, inflammation, and glomerulosclerosis in hyperglycemic rats, halting the progression of diabetic nephropathy [20]. In this study, LCZ696 was tested against DKD in the HFD/STZ rat model, a well-known model that mimics human type 2 diabetes mellitus, and its effects were compared with those obtained with valsartan as a comparison to examine if LCZ696 conferred any additional benefit over renin–angiotensin–aldosterone system (RAAS) blockade alone. The LCZ696 therapy effectively reduced the progression of DKD through the inhibition of inflammation and caspase-dependent apoptosis, as well as an increase in cellular antioxidants. Additionally, the current findings indicated that treatment with LCZ696 had nephroprotective effects, which manifested as a significant decrease in serum urea and creatinine and an improvement in renal functions. The diabetic rats exhibited clear injuries in the glomerulus and renal tubules, as evidenced by an enlarged glomerular space, obvious inflammatory infiltration, thickening of the glomerular capillary basement membrane, expansion of the mesangial matrix, tubular dilatation and disorder, and unclear tubular borders, as revealed by H&E and PAS staining. Importantly, LCZ696 treatment ameliorated HFD/STZ-induced histopathological changes. In addition to LCZ696’s nephroprotective effect, the aforementioned results confirmed renal dysfunction and established the DKD model successfully. The level of apoptosis markers Bax and caspase-3 were also dramatically reduced by LCZ696 therapy, while the expression of antiapoptotic Bcl-2 was elevated in the renal tissue. Additionally, a decrease in NFκ-B was linked to the restoration of the increased proinflammatory cytokines TNF-, IL-6, and IL-1 that were observed in LCZ696-treated rats.

Diabetes caused by HFD/STZ is a well-known model that closely resembles human T2DM [33]. Providing HFD to experimental animals along with a low dose of STZ causes insulin resistance and aberrant metabolic processes. Therefore, the present findings in this study were consistent with earlier work from our lab [26] as well as other studies [34] that have shown hyperglycemia and altered insulin secretion in HFD/STZ-induced animals. As a result, the HFD/STZ-induced rats in this study exhibited hyperglycemia and impairments in insulin secretion. It is interesting to note that administering LCZ696 to diabetic rats reduced hyperglycemia, demonstrating the drug’s capacity for glycemic control as previously reported [21,35]. In the current study, elevated serum levels of urea and creatinine, which are indicators of renal dysfunction, were seen in HFD/STZ-induced rats. These findings are consistent with those of Wahab et al., [33] who demonstrated impaired kidney function in type 2 diabetic rats. The serum levels of urea and creatinine in diabetic rats treated with LCZ696 significantly decreased, showing a considerable improvement in the kidney functional changes brought on by type 2 diabetes. Additionally, LCZ696 treatment restored renal impairment more effectively than valsartan. The findings of Rahman et al. [24] were consistent with those of the present study; LCZ696 was found to protect rats with type 2 diabetes with overt proteinuria against renal injury by decreasing the serum levels of urea and creatinine.

A critical factor in the onset and progression of diabetic kidney disease (DKD) is oxidative stress, which is exacerbated by an excess of reactive oxygen species (ROS) and a deficiency in antioxidant mechanisms [36]. Studies have shown that blocking or lowering the generation of ROS can stop renal damage caused by a variety of stressors [3,37]. In rats induced with HFD/STZ, we observed significant increases in TBARS levels, as well as significant decreases in the activity of GSH, SOD, CAT, GPx, and GST, indicating an overt state of oxidative stress. Similar findings were found in the kidneys of the experimentally induced type 2 diabetic rat model [38,39] with high lipid peroxidation, which may be related to an inadequate antioxidant system. However, in our study, treatment of HFD/STZ-induced rats with LCZ696 restored kidney TBARs levels and increased GSH, SOD, CAT, GPx, and GST activities, protecting them from oxidative stress. This effect of LCZ696 was stronger than that of valsartan. These results of our study indicate that LCZ696 is a potent antioxidant and free radical scavenger, which is important in mitigating the consequences of diabetes mellitus. Our results support earlier studies that found that LCZ696 can reduce oxidative stress in a variety of animal models, including diabetic cardiomyopathy [26,40], STZ-induced hyperglycemic rats [20], and the 5/6 nephrectomy rat model [41].

In addition to oxidative stress, inflammation is regarded as a prevalent aspect of chronic kidney damage and a crucial mediator in its development [42,43]. The constant generation of ROS during hyperglycemia causes uncontrolled oxidative stress, which in turn activates a variety of stress-sensitive signaling pathways, including NF-κB [44], whose activation leads to the increased expression of many gene products that cause cellular damage and is a crucial factor in the development of diabetes complications. Our findings, which were in line with earlier studies [45,46], showed that rats given HFD/STZ had considerably higher circulating levels of TNF-α, IL-1β, IL-6, and NF-κB as well as decreased IL-10. Contrarily, administration of LCZ696 to HFD/STZ-induced rats resulted in anti-inflammatory effects as shown by a considerable decline in TNF-α, IL-1β, IL-6, and NF-κB levels along with a significant increase in the levels of IL-10. One appealing fact in this study is that LCZ696 can reduce the inflammatory response induced by HFD/STZ, probably by inhibiting the NF-κB pathway. We have reported similar results in our previous work [20,26] and in other investigations [47] also reported that LCZ696 has anti-inflammatory capabilities which may be a key factor in its renoprotective potential against HFD/STZ-induced diabetic nephropathy. Additionally, LCZ696’s inhibitory effects on LPS-induced endothelium damage serve as further proof of the drug’s anti-inflammatory actions [48].

Numerous reports have shown that oxidative stress and inflammation brought on by hyperglycemia can cause apoptosis, damage the kidneys, and ultimately result in organ failure [44,49]. Therefore, one of the most important ways to stop programmed cell death in diabetic nephropathy is to suppress the elevated oxidative stress and inflammation. The present study showed that rats given HFD/STZ induced renal apoptosis. Immunohistochemistry analysis showed significant increases in apoptotic markers such as caspase-3 and Bax and decreased expression of antiapoptotic Bcl-2 in renal tissue of HFD/STZ when compared with the control. Similar to our findings, previous studies found that HFD/STZ could cause kidney tissue to undergo apoptosis by raising the expression of apoptotic markers such as caspase-3 and Bax [50,51,52]. Treatment of diabetic rats with LCZ696 significantly reduced the expression of pro-apoptotic factors such as caspase-3 and Bax and significantly improved antiapoptotic factors (Bcl-2) indicating the antiapoptotic potential of LCZ696. The observed attenuation in renal apoptosis after LCZ696 treatment may be a result of observed suppressions in oxidative stress and inflammation. These findings were supported by earlier research that showed LCZ696 to have antiapoptotic capabilities against diabetic cardiomyopathy [40], arsenic trioxide-induced cardiotoxicity [53], cyclophosphamide-induced testicular toxicity [54], and human umbilical vein endothelial cells (HUVECs) induced by oxidized low-density lipoprotein (ox-LDL) [55] by upregulating the suppressed Bcl-2 expression and downregulating the elevated Bax and caspase-3.

More importantly, LCZ696 therapy reduced oxidative stress and inflammation, which in turn shielded renal cells from injury and apoptosis, explaining why LCZ696 was more effective than valsartan in this study. The combined inhibition of the Ang II receptor and neprilysin by LCZ696 was more efficient than the Ang II receptor inhibition by valsartan alone, which may account for this protective effect [56]. The study’s limitations include the absence of a group receiving sacubitril alone since a selective neprilysin blocker with sacubitril would increase circulating levels of both Ang II and brain natriuretic peptide (BNP), which would then reverse the effects of the former [1].

In conclusion, our work shows that the dual inhibition therapy with LCZ696 had protective benefits against HFD/STZ-induced diabetic nephropathy by reducing oxidative stress and inflammation by regulating NF-κB and Bax/Bcl-2/caspase-3 signaling pathways. These findings support the clinical usage of LCZ696 in the future to maintain renal functioning in T2DM.

## Figures and Tables

**Table 1:** The effects of LCZ696 and valsartan treatment on serum glucose, insulin, creatinine, urea, and cytokine levels in HFD/STZ-induced diabetic rats.

Parameters	Control	HFD/STZ	Valsartan	LCZ696
Glucose (mg/dL)	109.9 ± 5.713	353.5 ± 14.75 ***	319.0 ± 1.631 ^#^	310.9 ± 1.908 ^##^
Insulin (ng/mL)	0.7128 ± 0.09232	0.6033 ± 0.05789	0.6741 ± 0.04930	0.6740 ± 0.03551
Urea (mg/dL)	9.974 ± 0.7861	41.54 ± 3.354 ***	37.11 ± 2.996	26.41 ± 2.133 ^##,+^
Creatinine (mg/dL)	8.172 ± 1.030	28.67 ± 1.406 ***	25.15 ± 1.379	18.23 ± 0.8938 ^###,++^
IL-6 (pg/mL )	169.6 ± 8.791	384.9 ± 24.35 ***	319.4 ± 14.16 ^##^	313.2 ± 15.81 ^##^
IL-1β (pg/mL)	86.16 ± 4.230	167.5 ± 4.082 ***	137.5 ± 7.706	99.78 ± 12.38 ^###,+^
TNF-α (pg/mL )	75.15 ± 3.576	149.5 ± 14.76 ***	106.3 ± 3.055 ^##^	93.93 ± 7.132 ^###^
IL-10 (pg/mL )	49.89 ± 3.222	26.44 ± 1.556 **	44.72 ± 3.777 ^#^	49.73 ± 5.450 ^##^

The data are expressed as the mean ± SEM, *n* = 6, *** *p* < 0.001 and ** *p* < 0. 01 versus control; ^#^ *p* < 0.05, ^##^ *p* < 0.01, and ^###^ *p* < 0.01 versus HFD/STZ; ^+^ *p* < 0.05, ^++^ *p* < 0.01 versus valsartan. Abbreviations: TNF-α, tumor necrosis factor-α; IL-1β, interleukin-1β; IL-6, interleukin-6; IL-10, interleukin-10.

## Data Availability

The data used to support the findings of this study are included within the article.
